# Healthcare Professionals' Views on the Use of Passive Sensing and Machine Learning Approaches in Secondary Mental Healthcare: A Qualitative Study

**DOI:** 10.1111/hex.70116

**Published:** 2024-11-25

**Authors:** Jessica Rogan, Joseph Firth, Sandra Bucci

**Affiliations:** ^1^ Division of Psychology and Mental Health, School of Health Sciences, Faculty of Biology, Medicine and Health, Manchester Academic Health Science Centre The University of Manchester Manchester UK; ^2^ Greater Manchester Mental Health NHS Foundation Trust Manchester UK

**Keywords:** artificial intelligence, interviews, machine learning, mental healthcare, passive sensing, qualitative, views

## Abstract

**Introduction:**

Globally, many people experience mental health difficulties, and the current workforce capacity is insufficient to meet this demand, with growth not keeping pace with need. Digital devices that passively collect data and utilise machine learning to generate insights could enhance current mental health practices and help service users manage their mental health. However, little is known about mental healthcare professionals' perspectives on these approaches. This study aims to explore mental health professionals' views on using digital devices to passively collect data and apply machine learning in mental healthcare, as well as the potential barriers and facilitators to their implementation in practice.

**Methods:**

Qualitative semi‐structured interviews were conducted with 15 multidisciplinary staff who work in secondary mental health settings. Interview topics included the use of digital devices for passive sensing, developing machine learning algorithms from this data, the clinician's role, and the barriers and facilitators to their use in practice. Interview data were analysed using reflexive thematic analysis.

**Results:**

Participants noted that digital devices for healthcare can motivate and empower users, but caution is needed to prevent feelings of abandonment and widening inequalities. Passive sensing can enhance assessment objectivity, but it raises concerns about privacy, data storage, consent and data accuracy. Machine learning algorithms may increase awareness of support needs, yet lack context, risking misdiagnosis. Barriers for service users include access, accessibility and the impact of receiving insights from passively collected data. For staff, barriers involve infrastructure and increased workload. Staff support facilitated service users' adoption of digital systems, while for staff, training, ease of use and feeling supported were key enablers.

**Conclusions:**

Several recommendations have arisen from this study, including ensuring devices are user‐friendly and equitably applied in clinical practice. Being with a blended approach to prevent service users from feeling abandoned and provide staff with training and access to technology to enhance uptake.

**Patient or Public Contribution:**

The study design, protocol and topic guide were informed by a lived experience community group that advises on research projects at the authors' affiliation.

## 1 Introduction

Approximately 10% of the world's population is living with a mental health difficulty [1]. Those who experience severe mental health difficulties, such as psychosis, can be at greater risk of poorer lifelong outcomes [2]. This is partly associated with the risk of relapse, which can be distressing for individuals and resultant treatment costs can be expensive [3]. Mental healthcare that supports long‐term treatment management and assessment is therefore important to reduce the risk of relapse and promote wellbeing [4]. However, a substantial gap persists between those needing care and those with access to care [5]. A global shortage of mental healthcare providers has cast doubt on the possibility of ever meeting the demand for mental healthcare through in‐person care alone [6].

Digital technology could play a significant role in addressing the challenges faced by services and people who experience mental health difficulties by offering flexible and tailored approaches to mental healthcare that are more accessible than those currently available [7]. Indeed, it has been suggested that digitising healthcare will support people to live independently, have more control over their lives and prevent health and social care needs from escalating [8]. The rapid growth in access to digital devices, which for the purposes of this article refers to all modern smartphones, smartwatches and other wearable devices that have sensors embedded, could augment traditional mental healthcare and bridge the gap between the need for treatment and capacity to deliver it [6]. Passive sensing allows for the continuous, unobtrusive collection of health data and can be used to understand an individual's activity levels, sleep and social engagement [9]. A large amount of raw sensor data can be collected this way and translated into markers of behaviour, emotions and mental states using machine learning, which is a branch of artificial intelligence (AI) that can be applied to large datasets to ‘learn’, identify patterns and draw inferences [10]. Machine learning methods can essentially detect when an individual's online footprint, generated from apps, social media and other personal monitoring devices, may indicate an impending risk of illness, self‐harm or even violence, thereby automating risk monitoring [11]. Service users can therefore track and collect clinically relevant data using their mobile or wearable devices and share this data with health professionals [12]. This can assist in the detection, diagnosis and treatment of mental health difficulties [13]. For example, raw sensor data captured about speech characteristics, location and activity can be transformed to derive high‐level behavioural markers around fatigue, sleep disruption and mood, which can be used to identify clinical states, such as depression [10]. Recommendations, feedback and reminders can then be delivered in‐the‐moment to inform users about their current state and how to improve it [14].

Bucci et al. suggest that theory‐driven digital interventions that monitor distressing experiences and provide near real‐time active management strategies could improve the speed and quality of recovery in psychosis over and above conventional treatments [15]. Digital interventions can also encourage autonomous motivation which, according to self‐determination theory, is facilitated through satisfaction of three basic psychological needs: autonomy, competence and relatedness [16]. When service users have these needs met in healthcare, they may be more engaged in treatment and experience better health outcomes [17]. In line with this, digital health interventions (DHIs) can empower individuals by facilitating self‐management, increasing self‐awareness and prompting the use of coping skills [18, 19]. Other potential benefits of digitally‐enabled care pathways include the collection and provision of objective measures that can supplement subjective self‐reports, which can be prone to recall bias [20]. Furthermore, traditional service delivery methods rely on service users seeking treatment themselves, but, mental health difficulties can hinder help‐seeking behaviour. Thus, digital health systems could identify individuals in need of support and accelerate their access to treatment [10].

Conversely, previous research has highlighted concerns about the potential for service users to interpret their behaviour data negatively, which could result in demotivation and frustration [18]. Concerns around data security and privacy issues are also widely discussed, with fears that access to a large amount of personal data could lead to confidentiality breaches [21]. In addition, it has been suggested that replacing direct contact with healthcare professionals with digital support could negatively impact the therapeutic relationship. This is due to reduced attendance to non‐verbal cues and loss of social connection provided by in‐person appointments [21]. Further concerns include the potential for clinicians to become reliant on digital health systems that use AI, which could result in misdiagnosis or missed diagnosis. This, in turn, could lead service users to lose trust in clinical decision‐making [22]. Previous research has also highlighted clinicians' concerns that integrating passively collected data and machine learning algorithms into clinical practice could increase their workload. Clinicians reported feeling ‘overwhelmed’ when presented with passive sensing data due to the huge volume of data generated by sensors [23] and concerns about the time involved in reviewing this data before and during sessions [24]. Clinicians have also expressed workload concerns about integrating this data into practice, the methods for receiving service user data, and their potential responsibility for monitoring data for risk issues [25]. A recent systematic review of 175 papers examining the barriers and facilitators of user engagement with DHIs for people with severe mental illness [26] identified several key facilitators. These included recognition of DHIs' advantages, a clear alignment between the intervention focus and service user needs, a simple and low‐effort digital interface, human‐supported delivery and device provision when necessary. Despite staff concerns about loss, damage or selling of equipment, studies reported only an 11% device loss rate. Barriers included intervention complexity, perceived risks, user motivation, discomfort with self‐reflection, digital poverty, symptoms of psychosis, poor compatibility with existing clinical workflows, fears that DHIs would replace traditional face‐to‐face care, infrastructure limitations and limited financial support for delivery.

Mental health clinicians' views on using digital devices to collect passively sensed data, which can then be used to generate machine learning algorithms for insights into an individual's mental health, may affect its adoption and usage [11]. For this reason, engaging and involving frontline staff in the design and rollout of digital approaches to mental healthcare is essential for ensuring utilisation and successful uptake [27]. Despite this, there is sparse research exploring the views of mental health staff on the use of passively sensed data collected from digital devices and applying machine learning algorithms generated from these data in mental healthcare. The aims of this research are twofold, to explore: (i) healthcare professionals' views and attitudes towards the use of digital devices, passive sensing technology and machine learning algorithms as applied in mental healthcare; and (ii) barriers and facilitators to incorporating digital healthcare systems into routine clinical practice. Qualitative methods using individual semi‐structured interviews will enable in‐depth exploration of this relatively unexplored area. This method will provide valuable insights into participants' experiences and perceptions of digital systems that utilise passive sensing and machine learning algorithms in mental healthcare [28].

## 2 Methodology

### 2.1 Design

This study used a qualitative design. Semi‐structured interviews were conducted with clinicians working in secondary mental health services (services for people with complex or long‐term mental health conditions that are beyond the scope of primary care) who approached the research team and were eligible to take part (*N* = 15). A predominantly inductive approach to analysis was adopted. Data was open‐coded, and meanings based on the interpretations made by interviewees were emphasised. However, the questions asked in the interview, although used flexibly, meant that deductive analysis was also employed to ensure that the open coding allowed for the identification of themes that were meaningful to the research questions posed. The analysis approach emphasised the importance of the researcher's subjectivity as an analytic resource and their reflexive engagement with theory, data and interpretation [29]. Questions were open‐ended with sequencing dictated by the flow of the exchange. Probes were used to aid further elaboration of responses. A critical realist epistemological position underpinned the research, recognising that an objective social world exists outside of people's constructions of it and that one's understanding of the social world is limited by one's experiences and position within it [30]. The study design, protocol and topic guide were informed by a lived experience community group that advises on research projects at the authors' affiliation. The group advised on topic guide development, including phrasing of questions and use of terminology and conducting qualitative interviews. In the development of this study, the consolidated criteria for reporting qualitative research (COREQ) checklist was consulted with regard to reflexivity and study design [31]. A working lay description of each of the relevant concepts (e.g., active symptom monitoring, passive sensing, machine learning) was given to help so that participants were working from the same reference point. This study was approved by the University Ethics Committee (Ref: 2021‐12959‐20908) and the Health Research Authority (21/HRA/5696).

### 2.2 Recruitment

Participants were recruited from secondary mental health services in the Northwest of England. Inclusion criteria were (i) experience working with service users in secondary mental health services; (ii) currently working in a clinical role and (iii) agree for the interview to be audio‐recorded. Service managers of relevant NHS Trusts were approached and information about the study was sent, including a participant information sheet and consent form to circulate to staff. The first author (J.R.) also offered to attend departmental meetings and contact details were provided, with participants invited to contact J.R. with any questions. All participants provided written informed consent and a final sample of 15 staff members took part.

### 2.3 Data Collection

All one‐to‐one interviews were conducted by the first author (J.R.) between June and November 2022, over an online platform (Microsoft Teams). Online interviews were offered to reduce demands on participants' time. Participants were asked to complete an anonymised demographic form. Interviews were audio‐recorded using an encrypted digital recorder and lasted between 30 and 60 min (*M* = 45). The interviews were centred on a topic guide (Table S1), which was created based on the research questions and existing research. Open‐ended questions were used to facilitate discussion around: (a) participant's personal use of digital devices; (b) digital devices in healthcare, including specific discussion around passive sensing and machine learning and (c) impact on role and practice. Early transcripts were discussed within the research team to reflect on the topic guide and allow refinement, such as further exploration of areas. For example, the topic of consent was raised and so prompts were added to explore this further.

### 2.4 Data Analysis

Interviews were transcribed verbatim, anonymised, given an identifying code and stored securely. Transcripts were read through whilst listening back to interviews to check for errors. Data analysis followed the recursive and iterative six‐stage thematic analysis protocol to facilitate coding and theme‐identification: (i) familiarisation with the data; (ii) generating initial codes; (iii) generating themes; (iv) reviewing potential themes; (v) defining and naming themes and (vi) producing the report [31]. Interview transcripts were read multiple times by the author (J.R.) to allow familiarity with the data. Initial codes were generated to summarise the relevant data content. Codes were grouped into potential conceptual themes (J.R.) and reviewed and refined by the research team (J.R., S.B. and J.F.) to ensure they were reflective of the original data, related to the aims of the research and sufficiently distinct to ensure they reflected differing theoretical concepts. Differences in opinion were managed through discussion and reviewing the data together. There was no attempt to determine intercoder reliability, as this can contradict the interpretive agenda of qualitative research [32]. Instead, quality assurance of coding, theme development and the final write‐up were guided by a tool for evaluating research quality [29]. Data collection was stopped when data saturation was reached [33]. NVivo qualitative data analysis software (NVivo version 12, 2018) was used to facilitate data management and analysis.

### 2.5 Reflexivity

Field notes and reflexive logs were kept throughout the interviews. Reflexivity is a process that addresses the subjective nature of research and increases the quality of research by enhancing the understanding of how the researcher's positions and interests impact all stages of the research process [34]. J.R. is a Trainee Clinical Psychologist and S.B. is an Academic Clinical Psychologist, both of whom have experience working in mental health services. J.F. is a UK Research and Innovation Fellow. Both S.B. and J.F. are principal investigators in clinical trials implementing digital health interventions and have extensive experience in conducting and supervising qualitative studies.

All authors utilise digital devices in their day‐to‐day lives. It is important to acknowledge that this may affect the analysis and interpretation of the data as the areas focused on may be issues the researchers found personally salient. Steps were taken to minimise the impact of this. JR ensured that interview questions were presented in an open and neutral way with no indication of the views of the research team. Participants were asked about their thoughts and were encouraged to explore both positives and negatives. JR also used reflective logs to consider how participants' responses influenced their own views and held this in mind throughout data collection and analysis.

## 3 Results

### 3.1 Participants

Overall, 15 participants took part in this study (3 males, 12 females). Participants were aged between 30 and 62 years (*M* = 45). Participants identified their ethnicity as White British (*n* = 13), British Pakistani (*n* = 1) and Anglo‐Arab (*n* = 1). Duration of experience working in secondary mental health services ranged from 3 years to 35 years (*M* = 12.7 years). See Table 1 for participant characteristics.

**Table 1:** Participant characteristics.

Participant number	Gender	Ethnicity	Profession	Time worked in secondary mental health services
P1	Female	White British	Clinical Psychologist	3
P2	Male	White British	Psychiatry Trainee	5
P3	Female	White British	Mental Health Nurse	20
P4	Female	British Pakistani	Consultant Psychiatrist	6
P5	Male	White British	Psychiatry Trainee	4
P6	Female	White British	Consultant Nurse	6
P7	Female	White British	Occupational Therapist	12
P8	Female	White British	Clinical Psychologist	7
P9	Female	White British	Mental Health Nurse	3
P10	Female	White British	Mental Health Nurse	17
P11	Female	White British	Principal Clinical Psychologist	12
P12	Female	White British	Mental Health Nurse	35
P13	Male	Anglo‐Arab	Consultant Psychiatrist	28
P14	Female	White British	Specialist Practitioner	21
P15	Female	White British	Senior Counselling Psychologist	11

### 3.2 Key Themes

**Figure 1:**
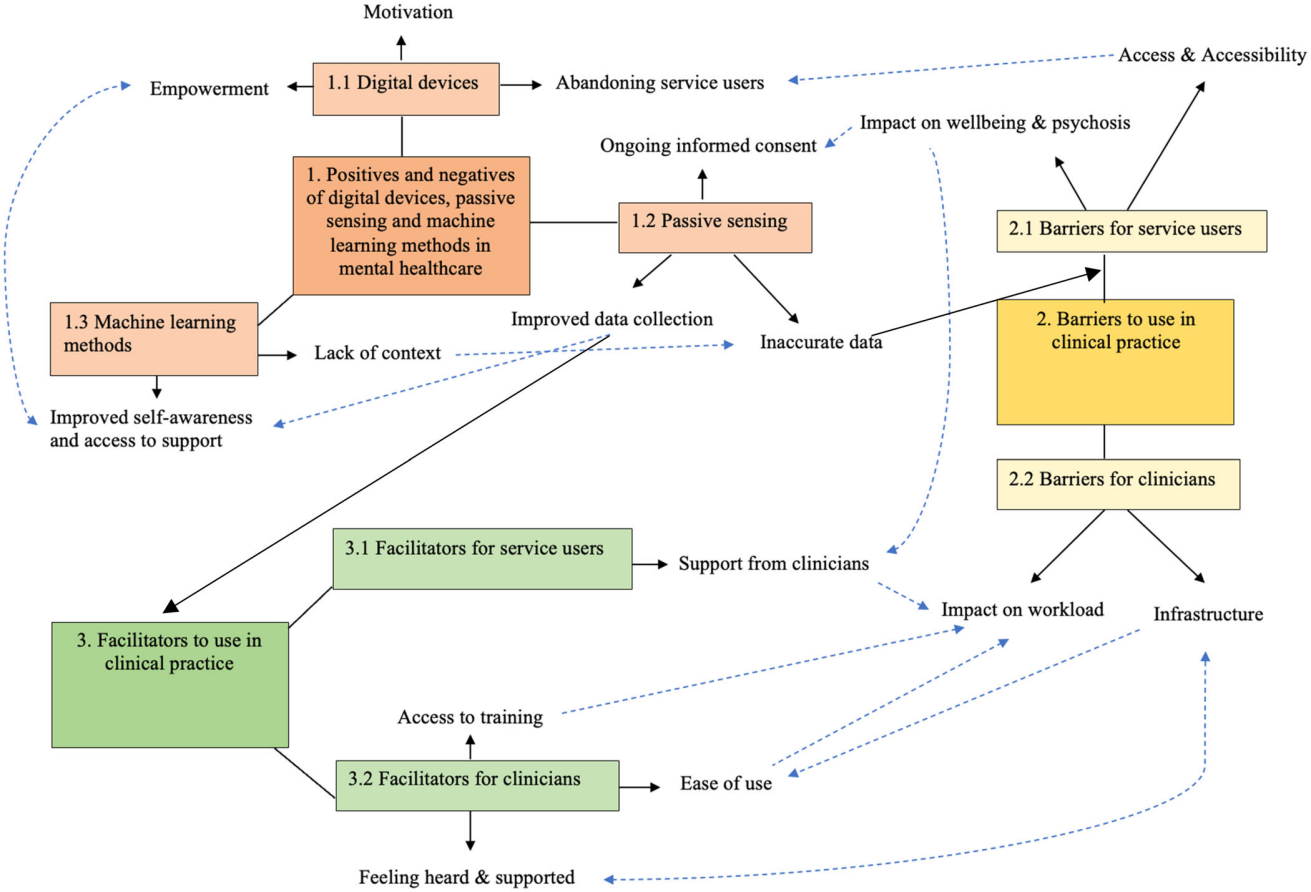
Relationships between themes and subthemes. Solid lines reflect direct links between concepts. Dotted lines reflect indirect links between concepts. Single‐direction arrows mean single direction; double‐ended arrows mean bidirectional relationship between constructs.

From the data, three distinct but inter‐related themes were identified: (1) positives and negatives of digital devices, passive sensing and machine learning methods in mental healthcare; (2) barriers to use in clinical practice and (3) facilitators to use in clinical practice. Seven subthemes were also identified. Themes, subthemes and relationships between them are represented in Figure 1 and summarised in Table 2.

**Table 2:** Summary matrix of main barriers and facilitators identified.

	Barriers to using passively collected data from digital devices in clinical practice	Facilitators to using passively collected data from digital devices in clinical practice
**Service users**	Access and accessibility	Support from staff
	The effect of receiving digitally delivered emotional and behavioural insights on wellbeing and psychosis	
**Staff**	Lack of supportive infrastructure	Access to training
	Impact on workload	Ease of use
		Feeling heard and supported

*Theme 1: Positives and Negatives of Digital Devices, Passive Sensing and Machine Learning Methods in Mental Healthcare*

During the interviews, participants were asked to consider digital devices, passive sensing and machine learning methods separately Consequently, Theme 1 is organised into digital devices, passive sensing and machine learning methods; participants highlighted both positive and negative aspects within each approach. We acknowledge, however, the overlap in concepts, as passive sensing is one function of digital devices like smartphones and smartwatches. However, we were concerned that issues around digital literacy might lead participants to conflate their views on different elements of digital approaches explored in this study. As such, we aimed to disentangle these elements to understand participants' views on each approach distinctly.

*Subtheme 1.1: Digital Devices*

Participants described both the positive and negative aspects to service users wearing or carrying digital devices with embedded sensors that collect mental health data and provide visual summaries, feedback and intervention strategies to service users. Positive aspects of digital devices centred on motivation and empowerment.

Using Digital Devices for Passive Data Collection Can Be Motivating

Participants said that setting goals, receiving alerts and getting behavioural insights from passively collected data might motivate service users to be more physically or socially active. This could be particularly beneficial for service users who are inactive, as their inactivity may be maintaining some of their difficulties:

…we have a lot of long‐term patients who have been in hospital a long time who don't exercise or very rarely get up or they're not doing much. So, I think it could be motivational for that patient group.

(Participant 5)

There was specific discussion around how devices that prompt engagement in activity and encourage motivation could be useful for people who have experienced psychosis, as they can have particularly poor health outcomes:

…people with psychosis, because of maybe the high doses of medication or the types of medication they're on or the nature of the illness, they've got a more sedentary lifestyle and we know from the research that their lifespan can be reduced by a number of years, can't it? And I think if we can encourage people to be more active… I think things like that are really helpful, because not everybody has somebody there to motivate them to do things.

(Participant 14)

It was further suggested that clinicians could support service users to connect with others who are using digital devices, to form groups in which they can encourage and motivate one another, which also facilitates social connection:

You know for patients you could even have little erm self‐help groups so you could set them up you know walking groups ‘off you go, go and test this equipment’, you know ‘come back, have a bit of a healthy competition’.

(Participant 6)

Using Digital Devices for Passive Data Collection Can Be Empowering

Participants discussed that service users accessing their data and intervention strategies offered to them via devices could be especially useful because it can be difficult when feeling distressed to remember coping strategies in‐the‐moment. A prompt via the device could remind service users what they could do to reduce their distress:

I suppose sometimes it's difficult to think in the moment especially if it's a moment when you're feeling more anxious or there's other stuff going on for you… to think actually what's going to be helpful for me in this moment… it might be harder.

(Participant 1)

Participants wondered if reminders of helpful strategies to manage distress could support service users to take more control of, and responsibility for, their wellbeing, which could be empowering:

…there's the proactive ‘I've got some control’ element, which is what we always want for people, is to have some autonomy and participation in their healthcare. I'm not having it done to me….

(Participant 12)

Participants also noted that when users self‐manage their mental health, it could have positive repercussions on services by reducing overall demand:

We know that mental health services are really stretched (…) So, I suppose this would help hopefully, for the right person who has the right plan, them to live independently and manage their own mental health.

(Participant 11)

Participants highlighted that a potential downside to using digital devices in mental healthcare is the unintended feeling of abandonment users might experience from their healthcare provider if this was used in replace of human contact.

Abandoning Service Users and Creating Inequalities

Participants cautioned that using digital devices might diminish the ‘humaneness’ of mental healthcare, potentially increasing feelings of isolation and abandonment by their healthcare provider if it results in less direct contact and interaction with clinicians:

… Because the risk of it is that people could feel abandoned to devices, a bit like robot world….

(Participant 12)

In addition, participants expressed concern about exacerbating inequalities in the mental healthcare system. They worried clinicians might inadvertently focus more on service users who are engaging with the digital technology, as they would receive more information from these users than from those who do not engage:

…I'm getting loads of information about the people that have consented and a lot less about people who have chosen not to have that then erm it would be… there's potential for more focus to go on the people you're getting the information about and less focus to go on the other people on your caseload.

(Participant 1)

Concerns were also expressed around creating a sense of abandonment once a service user was discharged from services if the device was taken away from them at this point. Participants felt that there could be potential negative consequences for a service user's recovery if the device was helping while they were engaged with their mental health provider, only to find the device was taken away once they were discharged from services:

Or do people only get access to this whilst they're in secondary care services? And then discharge…that feels a bit unfair and not very helpful for promoting recovery.

(Participant 11)

*Subtheme 1.2: Passive Sensing*

This subtheme summarises participant's views on the use of passive sensing in mental healthcare; that is, the passive collection of a large amount of data, which both service users and clinicians have access to. As in Subtheme 1.1, participants described both the positive and negative aspects to passively collected data for mental health. The positive aspect focused on enhanced data collection, while the negative aspects included challenges with ongoing informed consent and the accuracy of the data collected.

Improved Data Collection Requiring Less Effort

Most participants reported that they often recommend using an app or paper diary to track mood or behaviour in their clinical practice. However, they noted that the required effort, time and motivation can be a barrier for users. Participants recognised that a digital device, which passively monitors a user's mental health with minimal effort, might simplify data collection.

… they have to do less for us to collect the data, they don't have to open an app, click things, answer questions or ring us or text us. So, it requires less effort on their part if they carry their phone around that will tell us lots of information.

(Participant 4)

Participants noted that active monitoring might heighten distress and hypervigilance, as some mood or symptom‐tracking apps require significant time and effort for data input. In contrast, passive sensing is continuous and unobtrusive, potentially making it less stressful for users:

I've worked with people on sleep before where they keep a sleep diary, but then the amount of self‐monitoring people have to do… I feel like that's actually unhelpful for sleep, because it adds a level of stress to having to actually note the times and remember and write them down… if the wearables just doing it and they don't have to think about it, they can just put it out their mind and that data's still going to be collected, that would be really helpful.

(Participant 8)

In addition, participants believed that passively sensed data is more objective than subjective self‐reported data. As such, they viewed passive data collection as a better method for gathering information from service users since it reduces reliance on potentially biased and less reliable self‐reporting of symptoms and experiences. That is, participants perceived that service users might not tell the truth, lack insight or struggle with recollecting their thoughts, feelings and behaviours between appointments when actively inputting data into an app or paper diary, something that passively collected data can overcome:

…but yeah, it would be helpful, because obviously all our clients don't tell us the truth, do they?

(Participant 10)

It would help with their recollection of things as well, because as a consultant, I probably only see them infrequently.

(Participant 13)

It was also noted that, from the service user's perspective, collecting large amounts of data in near real‐time can validate their experience of psychosis over time, as it allows them to see and demonstrate their symptoms over time:

And it might be helpful for them because they can actually show like look, I'm not sleeping, like I'm not.

(Participant 9)

Negatives around the use of passive sensing in mental healthcare concerned ongoing informed consent and the accuracy (or inaccuracy) of data.

Concerns Around Privacy, Data Protection and Ongoing Informed Consent

A concern raised during discussions on passive sensing was around invading a service user's privacy, as clinicians might have access to vast amounts of information that the service user may not wish to share with their healthcare provider:

…they might not want their care team to know this information like they've got a right to privacy so erm if they feel like… it's a bit of an invasion of privacy.

(Participant 1)

Furthermore, concerns were raised about data protection. Participants were mindful about the need to consider the destination, storage, access and responsibility of passively collected data, especially when the data highlights issues requiring attention:

If we're collecting all this data, we need to be really clear about what will happen to the data. Will it be safe? Will it be secure? What actions will we take if there's a problem with the data?

(Participant 4)

The importance of capacity and informed consent was a prominent issue raised throughout interviews. Participants stressed that service users would need to know exactly what they are agreeing to when data was being collected passively over time. The passive nature of data collection can lead users to forget that their data is being recorded. This raises concerns about whether consent at regular intervals is necessary when digital devices are used for passive data collection, especially when this data informs mental health insights that impact clinical decisions:

…. the service users must know that that is happening…. That you are collecting the information, why you're collecting it and what it's going to be used for and if it's going to be shared. I think that those are necessary conversations.

(Participant 14)

Ongoing consent for passive data collection was deemed necessary, as a service user may become unwell and either withdraw or lose the capacity to provide consent:

I suppose that's why informed consent is going to be so important and thinking as well with psychosis that maybe capacity fluctuates… I think that's quite tricky….

(Participant 11)

The Accuracy (or Inaccuracy) of Passively Collected Data

Another issue raised was around clinicians trusting the data they receive due to concerns that passive sensing data is not always accurate and there may be negative implications if clinical decisions are made based on inaccurate data:

It sounds good in theory, my only thing is, particularly if we're using it to make clinical decisions, is to make sure that it's completely accurate because erm I am a bit sceptical in terms of how 100% definitely accurate it is.

(Participant 5)

Participants highlighted that data may be inaccurate if service users behave in a certain way to influence the data their clinician will receive. This could communicate a need, which might be useful, but could also mask needs:

…they could carry it or not carry it erm and that might be good or bad. They might use that as an indirect way of saying look erm (…) something's wrong or they might try and hide something and pretend everything's ok and pretend that they're going to sleep or walking around.

(Participant 4)

Participants wondered how they would manage discrepancies between self‐report and passive sensing data, particularly if the data is not completely accurate, as dismissing service user's claims could damage the therapeutic relationship and leave service users feeling invalidated:

…if the patient disagrees with the tech, we can't just go, ‘nope the tech knows best’, we have to temper that with…with patient experience because it is so important in maintaining the relationship, the patient relationship.

(Participant 2)

Participants highlighted how it would be difficult to have these conversations with service users as it might appear punitive, perhaps re‐enforcing a power imbalance between clinician and service user:

… because I'm not their mum. I don't want to go round telling them off because they've lied to me or you know not that they've lied, but you know they've not disclosed the whole truth.

(Participant 10)

*Subtheme 1.3: Machine Learning Algorithms*

This subtheme centres on participants' views on the use of machine learning methods in mental healthcare; that is, the idea that machine learning methods can be applied to passively collected data to identify patterns of potential worsening in an individual's mental health. As in Subthemes 1.1 and 1.2, participants described both the positive and negative aspects of using machine learning methods to develop algorithms that can be used in the context of an individual's mental healthcare. The positive aspect centred around the ability of a machine learning algorithm to identify trends and patterns in behaviours that could increase service users' and clinicians' understanding of an individual's difficulties and potentially spot early warning signs of worsening mental health, thus facilitating early assessment and intervention.

Improved Awareness and Access to Support

Participants felt that recognising trends and patterns in a service user's behaviour, relative to their own baseline, could be useful in practice. This approach could support both the clinician and the service user gain a deeper understanding of their difficulties and the factors that may be contributing to their onset and maintenance:

I like the idea that it would be their norm. So, you'd start by actually looking at what they do over a period, so hopefully we could capture them when they're relatively well (…) then you'd have a period of time where you'd capture data and then when they don't seem so well, you could then spot that trend, so it'd be compared to their norm.

(Participant 4)

This approach could help identify and raise service users' awareness of early warning signs, enabling them to access or seek support sooner or enabling clinicians to intervene earlier, thereby reducing the risk of relapse:

…we can identify when to intervene at a much earlier stage, which has lots of positives, positives for the patient, it could keep them out of hospital, you know, it could identify a medication review much earlier or you know simply support that needs to be put in place.

(Participant 6)

Participants thought that this approach could be especially beneficial for people who have already experienced an episode of psychosis. Since relapse can be particularly distressing for this group, being aware of early warning signs could help reduce the risk of hospitalisation:

…I think for people with psychosis, it could be really helpful for them to… you know for having a really good understanding of relapse and early warning signs and to notice them. It would be really helpful to have those notifications that kind of remind them of the things that would help or to go and see their GP.

(Participant 11)

Participants also expressed concerns about machine learning algorithms, particularly highlighting the lack of contextual data incorporated into algorithms, as well as the risk of diagnostic overshadowing.

Lack of Context

Participants highlighted that mental health is subjective, and participants questioned whether machine learning relies too heavily on objective, physiological data, potentially overlooking service user experience. That is, objectively collected data might not always align with a user's subjective experience. Participants suggested incorporating service users' input to better contextualise the data and ensure its accuracy:

…. the device thinks that your sleep is really different last night erm. . does this feel right from what your experience of your sleep was? Because sometimes it's totally wrong and if it's been totally wrong, you don't want it then flagging up alerts (…) we want to be led by our service user's perspective.

(Participant 8)

Concerns were also raised around diagnostic overshadowing, where behavioural changes might be mistakenly attributed to a mental health problem. In fact, these changes could indicate a physical health problem, leading to misdiagnosis or dismissing the underlying cause, potentially resulting in incorrect treatment:

So, I'll worry that someone's got this diagnosis of erm depression for example and they're really fatigued and they're sleeping loads and it's getting flagged up on a mental health app, ‘oh they must be having a relapse of low mood’, when actually it's a sign of cancer, a sign of something else that needs checking out….

(Participant 11)

*Theme 2: Barriers to Using Passively Collected Data From Digital Devices in Clinical Practice*

A prominent theme was barriers to using passive sensing in particular in mental healthcare. Participants highlighted separate barriers for both service users and clinicians and therefore we present these data separately. Participants did not identify machine learning as a significant barrier in clinical practice. This may be because they are unfamiliar with the difference between data capture and data use. Participant responses were more focused on the application of digital devices and passing sensing for managing service users' mental health and service provision, and less focused on machine learning, which involves developing algorithms and statistical models for complex tasks without explicit instructions.

*Subtheme 2.1: Barriers for Service Users*

Clinicians identified several perceived barriers to service utilising digital devices for passively collected data. These included limited access to digital devices, challenges in accessibility of data and feedback, and concerns about the impact of passive sensing technologies and data on wellbeing and symptoms of psychosis.

Access and Accessibility

Participants noted that service users may not have access to digital devices and if they were given access, there could be financial implications, such as charging the device or incurring data network costs to access the internet. Participants wondered if using digital devices to collect data passively to provide emotional and behavioural insights would actually be affordable for service users:

Some people don't have the resources, they don't have the money for internet connection, they don't have the money for a laptop or smartphone or iPad, whatever it is.

(Participant 14)

Participants also described their concern around providing service users with a digital device in the event they give the device away or lose or sell the device due to current cost of living demands or other pressures in their life that may lead to such behaviours:

… or even if they lost it. I shouldn't have said maybe ‘sell’ that was wrong of me, but you know what with the financial crisis that we're all heading towards apparently erm you know… you never know, do you?

(Participant 10)

Concerns around the stigma of owning and using a digital device to monitor one's mental health were also raised. Participants said that if devices were given to service users, they would have to be relatively ‘sleek’, ‘unobtrusive’ and not obvious that they were for a mental health problem to avoid stigmatising or revealing that the individual potentially has a mental health problem that requires monitoring:

…if people in the public cottoned on that people who generally were wearing this kind of device, if it was one that was initiated by the NHS, and could go ‘that means that you're being monitored for a mental health medication’ (….) erm you would have to have it as an incredibly generic device to avoid people just becoming aware of, ‘oh yeah, you must be ill’.

(Participant 2)

Participants further wondered about accessibility issues, such as eyesight, learning disability and dexterity, which may impact a service user's ability to engage with the technology and use it for its intended purpose. Age was also discussed, with participants wondering if older service users may struggle to use digital devices because they were less experienced with digital technology and the training needed might potentially be prohibitive:

I fear I'm being a bit stereotypical, but maybe… it's a big change for maybe an older adult population who never grew up with this kind of technology.

(Participant 11)

Indeed, it was felt generally that service users who have had less experience with digital devices may lack digital literacy and thus experience barriers to engagement:

If people have never used it, automatically they feel on the back foot because they haven't got that literacy.

(Participant 14)

On the other hand, participants recognised that introducing digital devices to less experienced service users could provide an opportunity to teach new skills and upskill service users:

…we should be skilling our patients up. So, if they don't know, let's use it as an opportunity to teach them, to show them….

(Participant 4)

The Effect of Receiving Digitally Delivered Emotional and Behavioural Insights on Wellbeing and Psychosis

Participants highlighted the potential impact of receiving emotional or behavioural insights from passively collected data on a service user's wellbeing and experience of psychosis, especially if they had been relatively inactive when the data was being collected. For example, if a service user received feedback about their inactivity, participants were concerned that this may further demotivate the service user and reinforce this inactivity rather than serve as a motivator to change:

…for some people it could be really demotivating and possibly negative when they're not achieving what they feel they should be achieving.

(Participant 12)

It was suggested that an increased focus on data itself could become a source of anxiety and create a fear of relapse, causing service users to over‐ or misinterpret a natural ebb and flow in mood as a sign of an impending relapse:

…. if you've got somebody who is really health conscious and who is really frightened of becoming very unwell again in the future, they can be maybe over monitoring themselves. So, they're taking normal fluctuations of mood for example as that's an early warning sign, something must be happening.

(Participant 14)

Indeed, finding the right balance between engaging with the data and becoming hypervigilant about it can be challenging and difficult to achieve:

…you're between a rock and a hard place, aren't you? Because you don't want people to be looking at it all the time and becoming obsessed with it and driven by it erm to completely ignoring it and not taking any notice of it.

(Participant 12)

Participants also shared their concerns for individuals experiencing paranoia, noting that devices that ‘track’ behaviour and provide feedback might be perceived as threatening and cause distress:

Someone with psychosis who maybe has some kind of paranoid thoughts or beliefs about people erm I don't think it would help to have this kind of tracking and know all these people know about them.

(Participant 11)

*Subtheme 2.2: Barriers for Staff*

Infrastructure and the impact of passively collected data from digital devices on workload were identified as barriers to the implementation of digital technologies and systems into clinical practice.

Lack of Supportive Infrastructure

Participants described how it would be challenging if guidance regarding digital technology and associated systems were provided without consideration of how use might differ between clinical teams or clinical settings. For example, the integration of passively collected data, which is then used to develop machine learning algorithms about an individual's mental state, might be applied differently in inpatient settings compared to community settings. Therefore, these systems need to be personalised rather than adopting a one‐size‐fits‐all approach:

I'm a bit of a negative nelly and I think it's just working for the NHS it's the experience of anything you roll out, it's just one size. It's never catered to individual teams you know that we… that we work with. It's sort of just like roll it all out and make it fit.

(Participant 10)

Participants also highlighted that NHS IT systems are disappointingly ‘slow’ and outdated, which may hinder or indeed prevent digital transformation. They also noted that the ratio of computers to clinicians is insufficient, potentially limiting access to the technology needed to use a digital health system in clinical practice:

…. at work we've got this RiO, we've got the systems and it's so slow compared to what it could be… you know it's a bit disappointing the systems in the NHS for me.

(Participant 3)

There's four or five desktops, but there's actually more staff than that so if we only loaded it onto a desktop device and they had to login… all the desktops we have are really slow erm it's really tricky getting erm that kind of access.

(Participant 4)

Concerns were also raised about device maintenance and ensuring they continue to work, as well as the insufficient access to IT support if a device failed and needed repair:

IT within the NHS is dreadful erm without being too unkind (…) erm but the level of IT that's required versus what we have is such a disparity er I can see that something like this… we would end up with a lot of faulty devices that don't work right or aren't recording or are recording wrong and no one who knows how to fix them.

(Participant 2)

Impact on Workload

Participants expressed concerns that using digital devices in practice could increase their workload, including tasks such as setting up devices, monitoring data and reviewing it with service users. As a result, staff may be resistant to adapting their practice to implement a digital care pathway:

Erm but you always get people in teams, don't you? That are resistant to that change, resistant to doing that little bit extra.

(Participant 6)

Participants felt that current demands, staff shortages, feeling under pressure and the overwhelming service demands would create even higher workloads, which are already unmanageable. These factors would likely prohibit the integration of digital approaches in clinical practice:

… erm we're short staffed, we're under pressure anyway. So, we're a bit in the negative zone at the minute, it's like well, when the hell am I going to do that? When can I do that?

(Participant 10)

Concerns were also raised around data flows and how clinicians would be alerted by an algorithm, for instance, notifying them of a service user's worsening mental state. This is particularly challenging when clinicians are already overwhelmed with multiple calls and emails related to user's clinical needs and care. Participants were curious as to whether clinicians would be required to act on alerts immediately and considered the impact this would have on their workload and clinical priorities:

…how the team receives that information and whether erm you know if it requires someone to act on that information like straight away or in the team …and the kind of the impact that might have on someone's workload….

(Participant 1)

Participants were also concerned about their responsibility to act, respond or defend themselves if something in the data flow indicated risk, especially if they did not have sufficient time to review the data flow and a risk indicator was missing. Participants felt that if this were to increase their workload, the outcome would need to justify the time invested:

…there was an indicator that would concern you… if you haven't seen that and then something happened, how would that stand up in a coroner's court?

(Participant 14)

And definitely that it's evident that the pros of getting this system erm are bigger than the cost to clinicians in terms of time and effort, because they already have little time to do anything else.

(Participant 11)

*Theme 3: Facilitators to Using Passively Collected Data From Digital Devices in Clinical Practice*

Participants discussed the factors that might encourage both service users and clinicians to engage with digital devices and passively sensed data in mental healthcare.

*Subtheme 3.1: Facilitators for Service Users*

Support From Staff

Staff support was identified as essential for facilitating service user engagement with digital approaches to mental healthcare. Participants felt that, as clinicians, they would play a key role in this process. They highlighted that a trusting relationship and a strong therapeutic alliance between the service user and clinician would be crucial to encourage buy‐in:

If you can develop that therapeutic alliance with the patient then… and if they've already seen some benefits of having involvement from the team (…) then they might be more open to trying novel types of technology.

(Participant 13)

Participants felt it was important for clinicians to ensure that service users have control over how they engage with digital approaches to mental healthcare. They emphasised that the process should be collaborative, promoting autonomy in their healthcare decisions:

…the collaboration around what this is for, we're not doing this to you. So, checking that that's kind of agreed (…) rather than, ‘we want you to do this’, and really kind of checking out that it is collaborative.

(Participant 12)

Having made this agreement with service users, documenting this in their care plan was viewed as further facilitating engagement:

As much as care plans are difficult to enforce, if a patient knows that part of the plan is that they wear this thing or they do this thing, they're often better at getting involved.

(Participant 2)

*Subtheme 3.2: Facilitators for Staff*

Access to Training

All participants highlighted the importance of high‐quality training. They suggested that training should help clinicians understand how to use digital devices, navigate associated systems, interpret data flows and integrate these into their practice:

Clinicians that are using it would need to understand the system, what the different options within the system were, how to get it all set up. I feel like there's obviously some level of training package needed.

(Participant 8)

Participants felt that presenting clinicians with an evidence base supporting digital approaches to mental healthcare could enhance engagement. This might help clinicians understand the rationale behind using digital technology in mental healthcare:

You could say well you know look there's evidence here that it really helps, that it's beneficial, there's been a study done and if you can provide evidence to say that it's… it has been helpful, I think erm team members would maybe be more supportive of it.

(Participant 9)

Furthermore, allowing clinicians to try the technology firsthand would enhance their understanding of a digital system, boost their confidence and skills, and enable them to effectively share this knowledge with service users:

It's always down to the training for me. So, the way around that for me would be to trial staff with it so they would have… they would know how it works because they're using it.

(Participant 3)

Ease of Use

Participants noted that for digital approaches to be effectively integrated into clinical practice, they must be user‐friendly, simple, efficient and straightforward. This ensures they do not disrupt workflows or take time away from other duties and responsibilities:

If it was something that just kind of worked very quickly and was minimal input from us, but if you kind of have to set something up for every service user and then you know, that was very time consuming I think that would be a problem.

(Participant 7)

Furthermore, clear and detailed guidelines and procedures must be established for clinicians. This will ensure clinicians know how to manage alerts and notifications about a service user's health and the appropriate follow‐up required:

So, if certain alerts come through, this is what you would do with it, you know like SOPS. You know you'd have to… you'd have to go through everything.

(Participant 3)

Feeling Heard and Supported

Participants felt that innovations such as digital approaches in healthcare often follow a top‐down process, without consultation with frontline staff or consideration of clinicians' views. There was a sense that this approach can be disappointing, disheartening and dismissive, ultimately devaluing the potential impact of such innovations on clinical practice:

Well, we never are consulted with anything. It all gets done upstairs and fed back down and… so, yeah, it's a bit just kind of this is how it is, and we have to get on with it.

(Participant 10)

Participants highlighted their desire to be meaningfully consulted during the development process and before implementation. Involving clinicians in this way would facilitate their engagement with digital technologies and systems, as it would allow them to inform and shape the system and feel that their input is valued rather than tokenistic:

I would say in terms of consultation and getting advice from teams before its even started and not the kind of half, ‘oh yeah, we really value your opinions’, when actually you know decisions are already made. So, proper meaningful engagement with patients and staff teams about how it's going to be done.

(Participant 11)

Participants also wanted opportunities to provide feedback on digital systems after their integration into clinical practice, perhaps through a built‐in mechanism. This would allow clinicians to report if tools are not functioning as intended or suggest adaptations to make them more useful, which in turn might increase their willingness to engage with digital technologies and systems:

I do find a lot of stuff is written without actually checking with the clinical staff and it's given to us as a kind of ‘here you go’ and we're like well that's useless, but we can tell you how to adapt it, (…) and actually having a direct line of communication. I would be a bit worried about you kind of going ‘well here you are, here's something, just get on with it….

(Participant 4)

## 4 Discussion

We investigated healthcare professionals' views and attitudes towards the use of digital devices, passive sensing technology and machine learning algorithms as applied in mental healthcare and the barriers and facilitators to incorporating digital healthcare systems into routine clinical practice. Three main themes were developed from the data: (1) positives and negatives of digital devices, passive sensing and machine learning methods in mental healthcare; (2) barriers and (3) facilitators to using passively collected data from digital devices in clinical practice.

Participants discussed that passive sensing technology could empower service users by giving them more control and responsibility for managing their daily mental health. Feeling empowered in this way could positively impact both service users and the broader mental healthcare system, as it may motivate service users to adopt healthier lifestyles and enhance health literacy. Indeed, an evaluation of the impact of digital inclusion in healthcare in the United Kingdom found that users who visited their GP less, had more confidence in using online health information and wider benefits were predicted for healthcare services and society as a whole [35]. Conversely, concerns were raised that delegating aspects of clinicians' roles to digital devices might make service users feel abandoned by technology, potentially harming the therapeutic relationship [36]. This issue is especially relevant for older people, those lacking technological literacy and access to digital devices as digital approaches to mental healthcare may marginalise these service users and exacerbate disparities [37]. To avoid digital exclusion, an evidence‐based digital inclusion strategy may be needed within mental health services [38]. Combining digital approaches with face‐to‐face interactions can prevent service users from feeling abandoned [27].

Clinicians generally felt that passive sensing technology allows for the unobtrusive collection of a significant amount of data without burdening service users. This is in line with previous research that has found passive sensing technology is less disruptive than keeping diaries or completing questionnaires [39]. Participants echoed concerns that have been raised previously around passive sensing data [40]. However, research suggests that using objective data alongside self‐report measures can improve the accuracy of detecting mental health difficulties, as self‐report can be biased for various reasons; combining them with objective data provides a fuller understanding of a person's challenges [41]. Furthermore, participants spoke positively about service users and clinicians receiving alerts regarding behaviour change, as this could allow earlier access to support, something previous research has also highlighted as valuable [24]. This approach is particularly useful for individuals who experience psychosis, as it can help reduce the likelihood of relapse and hospitalisation. This not only benefits the individual but also decreases demand and costs for services. Indeed, research shows that passive data is a better predictor of relapse than survey data alone [42]. However, participants expressed some discomfort around the collection of this data. There was a sense that passive sensing technology has the potential to invade the privacy of service users, as they will have less perceived control over the information they share with a healthcare provider. Concerns were also highlighted that those who experience psychosis may be prone to delusions and paranoia. Thus, a device that ‘tracks’ their behaviour might be seen as threatening [43].

Consent emerged as a prominent issue in the interviews. Participants highlighted the need for service users to fully understand what they are agreeing and consenting to and noted that this process must be ongoing due to potential changes in capacity over time. Research has shown that service users are generally comfortable sharing passively collected data with their clinician, but would not be comfortable sharing this data more widely (e.g. on social media sites) [14]. Therefore, as part of the consent process, the topic of data privacy and data protection is important to discuss, ensuring that service users know where their data goes, how it will be stored, who will have access to it and how long it will be stored for. Participants emphasised the need to offer choice and address barriers when obtaining consent, as some individuals may feel more confident with digital approaches than others. Fostering good relationships with service users, providing relevant information and clear reasons for change, and ultimately allowing service users to decide what is best for them are effective ways to encourage service user autonomy in their mental healthcare. This is in line with self‐determination theory, whereby supporting service users' autonomy, competence and relatedness is important to enhance adherence and health outcomes [16].

In relation to clinicians utilising digital devices that passively collect data and machine learning algorithms in practice, concerns were raised about the impact on clinician's workloads, with other studies highlighting the importance of this issue [18]. Participants felt that if digital devices and associated systems required extensive setup, monitoring and administrative work, along with receiving spontaneous alerts throughout the day, this would become unmanageable. Participants further shared a sense of scepticism that they would be given sufficient access to the technology and expressed that current systems would not be fit‐for‐purpose. Ensuring access to, and ease of use of, technology and systems will therefore be important, otherwise, this is likely to result in frustration and reluctance to engage [44]. Training is also important as some participants noted that they were older, lacked digital literacy and/or struggled with change. Indeed, digital technology and associated systems will only be useful if clinicians understand them and feel comfortable using them [23]. Although training was viewed as an additional time burden, participants highlighted ways to make it more effective. They suggested that staff try the technology themselves to gain firsthand knowledge to better support service users. Educating staff on the evidence base was also considered beneficial for understanding the advantages for practice and service users. Generally, it is important for training to build confidence and trust in systems [27].

In this study, there was an overarching sense that digital devices that enable passive sensing might result in new ways of doing things for both healthcare professionals and service users [45]. This was seen positively, as it recognises that digital technology is now integral to daily life and represents a step towards mental health services ‘catching up’ with physical health services. Further, participants identified ways in which they could use digital technology in practice to complement what they do or try new ways of working. Concerns appeared to be around uncertainty [40], with participants highlighting the importance of standard operating procedures, particularly around managing risk and data privacy [46]. Overall, healthcare professionals were open to any innovations that could improve the lives of service users. However, participants highlighted the importance of not abandoning service users to technology, and ensuring clinicians are not left to navigate these systems without support. Involving staff at every stage of development and deployment is crucial [23]; designing tools and systems *with* clinicians, rather than *for* them, helps humanise digital mental healthcare [45].

The study sample was heterogeneous, including staff of different ages and professional backgrounds, which allowed for a variety of views and attitudes to be captured. As staff worked across two large NHS trusts with different populations, including older adult populations, this allowed for a broad exploration of views and novel insights. However, we acknowledge potential bias in this study as staff who chose to take part may have held particularly strong views about the topic. In addition, participants' responses were specific to the clinical population they work with and the area in which they work (Northwest of England), and views/attitudes may be different elsewhere. However, participants were encouraged to consider the topic generally across mental healthcare. Although our study design, protocol and topic guide were informed by a lived experience community group that advises on research projects at our University, it might have been useful to consult multidisciplinary staff members during the development of the topic guide and to conduct a pilot interview to identify limitations. Rather, the topic guide in particular was refined during the research process to ensure sufficient exploration of views. For example, the topic of consent was a salient point raised during all early interviews. Prompts and follow‐up questions were added to facilitate further exploration of this area and capture additional views, such as if/why consent to the collection of passive sensing data might feel different to the collection of data that is actively collected.

During the interviews, clinicians primarily reflected on what mental health service users might perceive in using digital devices that passively collect data emotional and behavioural data. Whilst our intention was to also explore the use of machine learning algorithms in mental healthcare, participants reflected on this less, perhaps because they are unfamiliar with this approach and its current application in service provision. Research exploring service users' views directly is now needed to gain further insight. Furthermore, the views gathered in this study are hypothetical, reflecting attitudes before using digital systems and technologies in mental healthcare. Future research should explore actual acceptability, specifically clinicians' views after they have experience using these approaches in their practice [47].

Smartphone ownership among service users is high, making widespread roll‐out feasible. For example, a recent survey in the United Kingdom by members of our group found that 91.6% of psychosis service users own a smartphone. However, while wearable ownership is increasing, it remains lower (30.1%; Zhang et al., in press). Consideration must be given to ensuring people have the necessary equipment and engage with the technology long enough to collect sufficient longitudinal data for machine learning to develop accurate algorithms. Participants identified a number of potential benefits to using digital systems and technologies in mental healthcare. Staff noted that many people use devices such as smartphones and smartwatches in their day‐to‐day lives, and it makes sense to incorporate data generated from digital devices into practice. Various uses of the data were identified, including as a means of reflection, education, prompting conversations, and monitoring medication side effects. Furthermore, participants noted how service users could use these devices themselves to engage in healthier lifestyles, set health‐related goals, monitor and manage their mental health and set up support groups. Real‐time prompts to engage in coping strategies and highlight the risk of relapse were considered particularly useful for empowering service users, leading to broader positive implications for services.

## 5 Conclusions

As summarised in Table 3, our findings show the importance of consulting staff throughout the entire process, from design to implementation, and providing opportunities for feedback once devices and systems are introduced. Training is crucial, with several suggestions made to ensure it is engaging and meaningful for staff. Staff reflected on past experiences with new systems, recalling feelings of overwhelm and neglect, highlighting the need for sufficient training and ongoing support to be in place. Furthermore, systems must be fit‐for‐purpose and must not introduce new or additional barriers. Digital systems must also adhere to strict data management procedures to ensure data security and safety, with clear guidelines to help staff feel comfortable and confident when obtaining informed consent from service users.

**Table 3:** Recommendations based on study findings.

Impact on the therapeutic relationship	
	Start with a blended approach, combining face‐to‐face contact with digital device usage. This can help reduce feelings of abandonment and provide opportunities to address problems together.
	Consider ways to reconcile discrepancies between self‐reported and objective data to preserve the value of personal experience and the therapeutic relationship.
**Impact on staff**	Involve staff at every stage, from development to deployment. Providing comprehensive training that addresses concerns is essential. Ongoing and accessible support is crucial, especially for those with low digital literacy.
	Allowing staff to test the technology and review evidence of its practical benefits will enhance their engagement.
	To ensure staff are well‐prepared, standard operating procedures are needed and must be clear, especially for managing risk issues.
	To minimise significant increases in workload, systems should be fit‐for‐purpose and have efficient data flows.
	To ensure the safety and security of service user data, digital devices and systems must comply with stringent data management procedures that are clearly defined to help staff feel confident when obtaining informed consent.
**Impact on service users**	
	Ensure devices are user‐friendly and provide information in accessible formats, such as audio and video, not just text.
	Regularly review service users' experiences with the devices to evaluate their impact on wellbeing and address any issues/difficulties that may arise. This also provides an opportunity to re‐evaluate consent if circumstances change.
**Equity**	Make plans for those who choose not to use digital tools to ensure equitable healthcare.
	To address digital exclusion for service users, consider loaning phones or wearable devices to those without access. Develop plans for managing lost, stolen or broken devices, and inform service users about any associated costs, such as charging and using Wi‐Fi usage.

## Author Contributions

**Jessica Rogan:** methodology, data curation, investigation, validation, formal analysis, project administration, writing–original draft, writing–review and editing. **Joseph Firth:** methodology, supervision, writing–review and editing. **Sandra Bucci:** conceptualisation, methodology, investigation, validation, supervision, funding acquisition, writing–review and editing.

## Disclosure

The views expressed are those of the author(s) and not necessarily those of the NIHR or the Department of Health and Social Care.

## Conflicts of Interest

S.B. is a director and shareholder of CareLoop Health Ltd, a University of Manchester start‐up to develop and market digital solutions for mental health problems, currently in schizophrenia and postnatal depression.

## Supporting information

Supporting information.

## Data Availability

The data that support the findings of this study are available on request from the corresponding author. The data are not publicly available due to privacy or ethical restrictions.
