# Supplementary material for: Healthcare Professionals' Views on the Use of Passive Sensing and Machine Learning Approaches in Secondary Mental Healthcare: A Qualitative Study
Source: Health Expect. 2024 Nov 25;27(6):e70116. doi: 10.1111/hex.70116 (PMC11589162; doi:10.1111/hex.70116)
Supplement: Supplementary file 1 — Supporting information. [file HEX-27-e70116-s001.docx]

**Supplementary Table 1.**

**Introduction**

Introduce self, welcome and thank participant. Ensure comfortable, have privacy etc.

**Informed Consent**

Ensure participant has given written informed consent and still wishes to take part.

**Clarification of the purpose and length of the interview**

We want to get your views on the use of passive sensing and machine learning techniques in mental healthcare. The interview should take up to 1 hour, but we can take a break any time you want to. Reminder that the interview is audio recorded, participation is voluntary, and you can withdraw at any point.

**Clarification of anonymity and confidentiality**

As discussed, I will record our conversation. It will then be written up and given an anonymous number. All identifiable information will be removed. The written-up document will be stored in a password protected computer file, that only the researchers will have access to. Quotes from the interview might be used when we write the research up and publish; however, they will not be identifiable. Are you happy to continue?

**Interview**

General use of digital devices. Explore participants view of digital devices more generally and their feelings about this:

Please can you tell me a little bit about your current use of digital devices, such as smartphones, wearable devices and so on?

- *What is it that you use (smartphone/wearable devices)?*
- *How long?*
- *How often?*

What is your general view about digital devices?

PROBE if needed: *some might say they find digital devices enjoyable and helpful, whereas others might describe them as annoying or addictive ....*

Digital devices & health monitoring. Explore participants use of digital devices in health monitoring and understanding of the technology involved:

Do you use your digital devices yourself for health purposes?

- Prompt - *Counting steps, sleep routine, setting goals*
- *How do you find this?*
- *Is it helpful / unhelpful?*
- *What made you start doing this? If you started but stopped, what made you stop doing this?*

Interviewer to provide definition of passive sensing and machine learning, and a brief overview of applications to digital devices in healthcare, with examples:

*Smartphones and wearable devices continuously collect data from the user, without any user effort or impact, through what is known as "passive sensing". So,_for example these devices can track physical activity, social engagement, and sleep. This results in a lot of data being collected. Using machine learning, which is a type of Al, patterns in behaviour can then be 'learned' and so changes in behaviour identified. So, for example, if a person usually achieves a certain number of steps per day and makes a certain number of phone calls, they can be alerted if their behaviour seems different to their usual pattern. This is applicable to mental healthcare, in that changes in behaviour may be associated with relapse and so a person can be alerted to this, and therefore seek support as soon as possible. Advice and support can also be offered via the digital device used.*

- What do you think about this?
- Have you come across/recommended anything like this to service-users in your clinical practice?

*How do you find doing this? What has the feedback been like?*

*How does it support your practice/intervention?*

- What do you think about digital devices that use passive sensing technology being used in mental healthcare?

*Any concerns/benefits to practice?*

- What do you think about passive sensing technologies being used to monitor symptoms of psychosis specifically?

*How might it benefit service users? Do you have any concerns?*

*How do you think service users will find this? (Anticipated problems/benefits)*

- What are your thoughts on machine learning methods in this context?

*Any concerns /benefits?*

- What do you think about interventions being delivered via digital devices?

*How do you think service users will find this?* (Anticipated problems/benefits)

Healthcare professionals / clinical practice; Explore participants views of facilitators/barriers

- What do you see your role to be in this?
- What implications might this have on routine clinical practice?

*How might this technology be embedded into current interventions?*

- Do you see there being any barriers to using this type of technology in mental healthcare for people with psychosis?
- What might help?

Ending

Is there anything else you would like to tell me that we have not talked about, but might be helpful for me to know?

• Thank participant for taking part

• Explain what will happen now and how this information will be used

• Offer to provide summary of the findings when available.
